# Structure and Dynamics Guiding Design of Antibody Therapeutics and Vaccines

**DOI:** 10.3390/antib12040067

**Published:** 2023-10-18

**Authors:** Monica L. Fernández-Quintero, Nancy D. Pomarici, Anna-Lena M. Fischer, Valentin J. Hoerschinger, Katharina B. Kroell, Jakob R. Riccabona, Anna S. Kamenik, Johannes R. Loeffler, James A. Ferguson, Hailee R. Perrett, Klaus R. Liedl, Julianna Han, Andrew B. Ward

**Affiliations:** 1 Institute of General, Inorganic and Theoretical Chemistry, University of Innsbruck, Innrain 80/82, A-6020 Innsbruck, Austria; 2 Department of Integrative Structural and Computational Biology, The Scripps Research Institute, La Jolla, CA 92037, USA

**Keywords:** antibody, structure prediction, antibody structure determination, molecular dynamics, X-ray crystallography, NMR, cryo-EM, vaccine design, special formats

## Abstract

Antibodies and other new antibody-like formats have emerged as one of the most rapidly growing classes of biotherapeutic proteins. Understanding the structural features that drive antibody function and, consequently, their molecular recognition is critical for engineering antibodies. Here, we present the structural architecture of conventional IgG antibodies alongside other formats. We emphasize the importance of considering antibodies as conformational ensembles in solution instead of focusing on single-static structures because their functions and properties are strongly governed by their dynamic nature. Thus, in this review, we provide an overview of the unique structural and dynamic characteristics of antibodies with respect to their antigen recognition, biophysical properties, and effector functions. We highlight the numerous technical advances in antibody structure prediction and design, enabled by the vast number of experimentally determined high-quality structures recorded with cryo-EM, NMR, and X-ray crystallography. Lastly, we assess antibody and vaccine design strategies in the context of structure and dynamics.

## 1. Antibody Structure Characteristics

**Figure 1:**
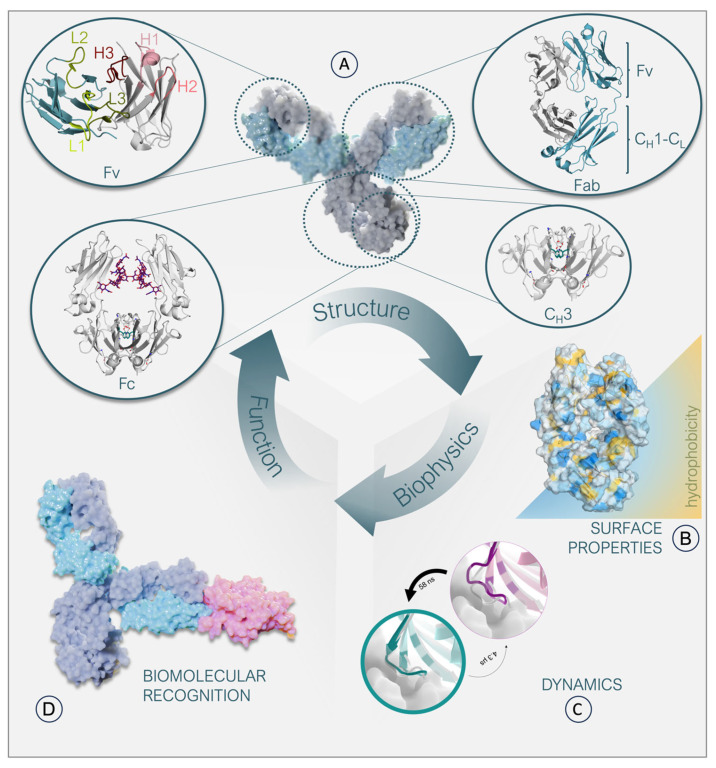
Relationship between structure, biophysical properties, and function of an IgG antibody. (**A**): Surface representation of an antibody together with structural depictions of its individual domains. The heavy chain is colored gray, and the light chain is colored light blue. The C_H_2-C_H_2 interface glycan interactions are colored pink. Amino acids constituting the hydrophobic core in the C_H_3-C_H_3 interface are colored light green. The Fv contains the CDR loops, which are highlighted in shades of green and red, respectively. (**B**): Representation of a Fab surface, color-coded based on its hydrophobicity. Hydrophilic patches are colored blue, while hydrophobic patches are colored yellow. (**C**): Depiction of two distinct loop states, with the more probable state colored in cyan and the less probable state in purple. The thickness of the circles indicates the probability of each state. The transition times between the two states, which can occur at the nanosecond or microsecond level, are indicated by the thickness of the arrows. (**D**): Surface representation of an antibody-antigen complex. The heavy chains of the antibody are colored dark blue, and the light chains are colored light blue. A model antigen is represented in pink.

Antibodies and related formats have become enormously important as biological drugs in recent decades, with more than 120 therapeutic antibodies approved by the US Food and Drug Administration (FDA) for use in humans [1,2]. The vast majority of approved therapeutic antibodies share the canonical IgG architecture, i.e., a symmetrical Y-shaped structure (Figure 1A,D). The modular anatomy of an antibody has inspired numerous engineering efforts to optimize and fine-tune the respective biophysical properties and increase their therapeutic potential. The classic IgG structure is composed of two identical heavy chains as well as two identical light chains [3]. The stem of the antibody is built of two analogously paired heavy chains that form the crystallizable fragment region (Fc), comprising the C_H_3-C_H_3 and the C_H_2-C_H_2 dimers. The C_H_3 domains are directly involved in interactions with cell surface receptors, such as the neonatal receptor [4,5]. The C_H_2-C_H_2 dimer differs from the other interfaces as it contains glycan interactions in the interface instead of forming direct protein-protein contacts (Figure 1A). The arms of the “Y” are heterodimers by nature, each consisting of a paired heavy and light chain. As the arms are responsible for antigen binding, they are called antigen binding fragments (Fab). The Fab can be further subdivided into a constant and variable domain (Fv). The variable fragment comprises six hypervariable loops, which are mainly involved in antigen recognition, called the complementarity determining region (CDR) loops (Figure 1A) [3]. The highest diversity in length, sequence, and structure is concentrated on these CDR loops, which pose challenges in predicting antibody structures, their developability as tools or therapeutics, and in elucidating the antibody-antigen recognition mechanism. While less prominent, sequence changes in the framework region (FR) can also have significant consequences for biophysical properties and antigen binding (Figure 1B) [6,7,8,9,10,11]. For example, the relative orientation of the variable domains (V_H_-V_L_) influences the shape of the antigen binding site, deemed the paratope (Figure 1A,D) [12,13].

## 2. The Ig-Fold

**Figure 2:**
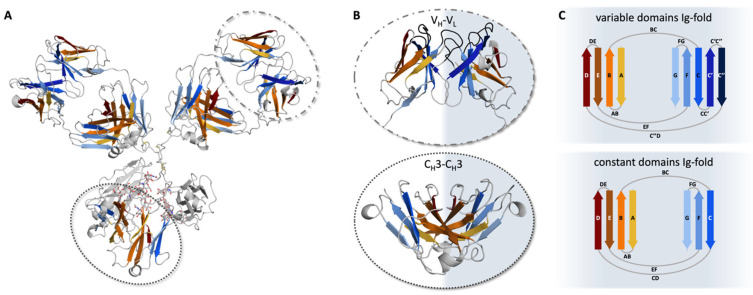
Pairing preferences of the Ig-folds in antibodies. (**A**) Crystal structure of an IgG antibody (PDB: 8TP9) [21] in cartoon representation. In orange and blue, the two beta-sheets of the Ig-fold are represented, respectively. The gray-shaded regions illustrate loops, or C_H_2-C_H_2 domains. V_H_-V_L_ and C_H_3-C_H_3 domains are highlighted by dashed and dotted circles, respectively. (**B**) V_H_-V_L_ (top) and C_H_3-C_H_3 (bottom) dimers are shown as cartoons. The CDR loops in the Fv are shown in gray, while the framework is colored based on the respective sheets, according to (**A**). (**C**) Schematic depiction of the Ig-fold. Variable domains (top) consist of two β-sheets, one with four (D, E, B, and A) and the other with five strands (C″, C′, C, F, and G). The constant domains (bottom) are also made up of two β-sheets, again one with four (D, E, B, and A) and the other with three strands (C, F, and G).

The individual antibody domains are characterized by the evolutionarily conserved immunoglobulin fold (Ig-fold; Figure 2A) [14]. This fold is common among various proteins covering diverse biological functions [15,16]. Each Ig-like domain of an antibody contains approximately 100–110 amino acids, forming two anti-parallel β-sheets stabilized by a buried intramolecular disulfide bridge [14]. The Ig-folds between constant and variable domains vary. Both include a similar β-sheet consisting of four β-strands (A, B, D, and E). The second β-sheet varies with the constant domain consisting of three β-strands (C, F, and G), while the variable domain features five strands (C, C′, C″, F, and G; Figure 2B,C). While the second β-sheet builds up the V_H_-V_L_ interface, the pairing of the constant domains (C_H_1–C_L_ and C_H_3–C_H_3) is facilitated by the first β-sheet (Figure 2C) [3,17,18,19]. Due to the assembly of the four chains, four to five interfaces are formed depending on the isotype of the heavy chain (Figure 2A) [20]. Generally, the chains belonging to the human IgG structure dimerize, forming either heterodimers, e.g., V_H_–V_L_ and C_H_1–C_L_, or homodimers (C_H_2–C_H_2 and C_H_3–C_H_3).

## 3. Antibody Structure Prediction

Acquiring high-resolution structures of an antibody of interest can be a laborious and resource-intensive task. Therefore, antibody structure prediction can significantly inform and expedite the antibody engineering process [22]. Accurate prediction of protein structures from their amino acid sequences is a critical challenge in computational chemistry [23,24,25]. Precise models could greatly enhance our comprehension of the physical and biological properties of existing therapeutic antibodies and facilitate the search for superior binders, thus positively influencing the development of future therapeutics [3,26].

The structure of most parts of an antibody, including the constant regions and the framework of the variable regions, is highly conserved [27]. Therefore, it is rather straightforward to model. However, the CDR loops in IgG-like antibodies exhibit significant structural diversity [28,29]. Where five of the six CDR loops can be represented by a set of canonical cluster conformations [30,31,32], the CDR-H3 loop has proven to be difficult to model accurately [29,33]. Furthermore, the multimeric structure of antibodies, where multiple non-covalently bound domains interact to form the complete antibody, makes it paramount to include the orientation of these domains [33,34,35,36]. For these reasons, the main conundrum in antibody structure prediction is that the antigen binding site, often the primary region of interest, is also the hardest to model [37]. Over the years, various methods have been developed to tackle this problem [26,38,39,40,41].

Template-based approaches, such as homology modeling, use the structural information from previously measured protein structures to build a model for a given sequence. Although these methods yield good results for most parts of the antibody, including the five canonical CDR loops, they are limited by the availability of structural data, particularly for antibody-like constructs such as bispecific antibodies or nanobodies. Hybrid methods, such as Rosetta antibody [42] or ABodyBuilder [43], instead include ab initio prediction methods to generate better loop ensembles, fill the gaps of missing data, and improve the modeling of uncertain regions. Recent, exhaustive reviews offer an overview of the vast array of available methods [26,38,39,40,41].

For several years, artificial intelligence-driven methods have advanced in predicting protein structures from their amino acid sequences. However, available protein structure prediction tools, such as AlphaFold2 [44,45], are less reliable for predicting the structures of CDR loops and multimeric proteins like antibodies [46]. Recent models tailored to describe CDR loops, such as DeepAB [47], IgFold [48], or ABlooper [49], have shown promising results in predicting the structures of CDR loops with greater accuracy. As highlighted by recent reviews, this is a rapidly evolving field of study set to revolutionize antibody in silico design [40].

Future tools may be further improved by incorporating information on the dynamic nature of the protein, as a description through a single structure may not capture all relevant properties. Especially the CDR loops are known to be notoriously flexible, and property prediction might therefore suffer from insufficient sampling of their structural ensemble. In addition, while most applications aim to model antibodies close to the crystal structure, these might be distorted compared to the structure in solution, which can be especially problematic for paratope predictions [50,51]. Including information on the accessible structural ensemble in solution through experimental methods such as NMR spectroscopy or cryo-EM as well as through computational methods such as molecular dynamics simulations may provide valuable insights. 

It is imperative to exercise caution and thoroughly scrutinize the generated models while employing any modeling approach. In a late comparison of various antibody modeling tools, unphysical inaccuracies were observed, which may affect the antibody’s predicted properties. [52] Notably, a comprehensive comparison of the available antibody modeling tools, including the new artificial intelligence-driven methods, is missing. As the last Antibody Modeling Assessment took place over 8 years ago [22,53,54], it might be time for another large-scale blind study assessing the current state and helping address the existing challenges in antibody 3D structure modeling.

## 4. Antibody Dynamics

**Figure 3:**
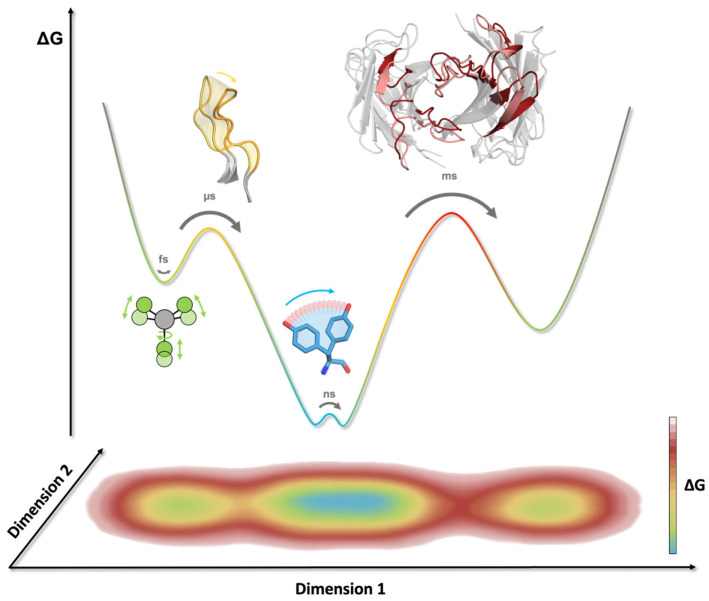
Timescales of conformational rearrangements in antibodies. The energy barriers correspond to the timescale required to overcome and assess different conformational states. A 1D representation of the free energy landscape (top) and 2D projection highlight the multidimensional character of certain movements and reaction coordinates. Bond vibration, rotation, and stretching can be observed on the femtosecond (fs) timescale (indicated in green), while sidechain motions can occur within the nanosecond (ns) timescale (highlighted in blue). CDR loop rearrangements take place on the micro- to millisecond (µs/ms) timescale (colored in yellow), whereas transitions between different paratope states can occur on the millisecond (ms) timescale (indicated in red).

Protein dynamics are a central driving force in all biological processes. Ultimately, protein functions are governed by their dynamic nature, and the same applies for antibodies (Figure 1C) [55,56]. Molecular dynamics simulations allow the characterization of functionally relevant structural rearrangements in atomistic detail as a function of time [57]. The high dimensionality of the conformational space of an antibody leads to a large and rugged free energy surface [55]. The depth of an energetic minimum reflects the probability of a specific conformational state. To transition from one state to another, the system needs to overcome the energetic barrier that separates them. Therefore, the height of this barrier determines the probability of a transition, i.e., the timescale for a conformational change to occur [50]. Thus, resulting from the multidimensionality of the energy landscape representing an antibody, different rearrangements can occur on different timescales. While subtle movements, such as bond vibrations, side-chain fluctuations, and variations in the interdomain orientations, are separated by low energy barriers and can therefore be observed on the nano-to-microsecond timescale, large loop rearrangements in the binding site can take milliseconds or even longer (Figure 3) [50,56].

Because the antibody scaffold can recognize and accommodate diverse antigens with distinct motifs, Pauling and Landsteiner proposed in the 1940s that antibodies follow the concept of conformational diversity. In other words, a single antibody can adopt various conformations and potentially recognize multiple antigens, thereby directly increasing the size of the antibody repertoire [58,59]. This idea of having an ensemble of pre-existing antibody conformations in solution out of which the functional ones are selected and expanded is in line with the conformational selection paradigm. In agreement with these hypotheses, the antigen binding site exists as multiple interconverting paratope states, revealing correlated CDR loop rearrangements that can result in shifted interdomain orientations [11,13,60,61]. The dominant antibody paratope state in solution has been frequently pointed out to coincide with the binding competent conformation, while unbound crystal structures can be distorted by crystal packing effects [28,62]. Additionally, it has been demonstrated that antibody-antigen docking can profit from incorporating different antibody paratope states and their respective probabilities [63]. Another critical aspect influencing and co-determining the conformations and properties of the antigen binding site are specific antibody framework residues. The importance of non-CDR loop amino acids should not be omitted, since up to 22% of residues that can interact with antigens fall outside the traditionally defined CDR loops [6,7,8,9,10,64,65]. Various studies have structurally and dynamically characterized the role of framework mutations on the CDR loops and the relative V_H_–V_L_ interdomain orientations and showed that a single-point mutation in the framework can influence antibody affinity and specificity. In particular, residue H71 located in the HV4 loop (Chothia nomenclature) determines the canonical conformation of the CDR-H2 loop and consequently affects the shape of the paratope [6,7,8,9]. Even the low-population states that occur more frequently near hydrophobic surfaces can play an important role in understanding processes such as aggregation or chemical modifications. In fact, these interactions with hydrophobic surfaces can result in a population shift towards more hydrophobic conformations, which are more likely to aggregate. Thus, accounting for the high conformational diversity of antibodies by considering them as ensembles in solution can advance the antibody development process, as the identification of potential liabilities and optimization of biophysical properties, such as hydrophobicity and electrostatics, can be facilitated (Figure 1B) [66,67,68]. 

Other critical aspects in antibody dynamics and engineering are the interdomain orientations (V_H_-V_L_, C_H_1-C_L_) and the elbow angle [69,70,71]. The majority of the interdomain movements have been reported to occur in the low nanosecond timescale (<10 ns). Additionally, for the V_H_-V_L_, it has been shown that the slow components of the motions (>10 ns) correlate with CDR-loop rearrangements [12,13,50,72]. In line with the surprisingly fast V_H_-V_L_ interdomain dynamics, we also find fluctuations in the same timescale for the elbow angle, which increase the variability of the whole Fab, enhancing the bivalent binding of antigens [13,73]. The elbow angle is defined as the angle between the pseudo-2-fold axes relating V_H_ to V_L_ and C_H_1 to C_L_. Previous studies have demonstrated that naturally occurring forces as well as antibody engineering approaches influence the dynamics of elbow and interface angles [6,13,60,72,73,74]. For example, varying one single so-called Vernier-zone residue (the region of the framework anchoring the CDR loops) or using a different combination of germline heavy and light chains can alter not only the CDR loop conformations but also the V_H_-V_L_ interface orientations [6,60]. Additionally, the type of the light chain has been determined to affect elbow and interface orientations, as λ-light chain antibodies reveal shifts and higher diversities in possible elbow V_H_-V_L_ and C_H_1-C_L_ angles [13,71]. Furthermore, maintaining a specific orientation of the variable domains has been shown to be important during humanization processes to preserve the antigen-binding properties [13,75]. 

## 5. Antibody-Antigen Recognition and Structure Guided Vaccine Design

**Figure 4:**
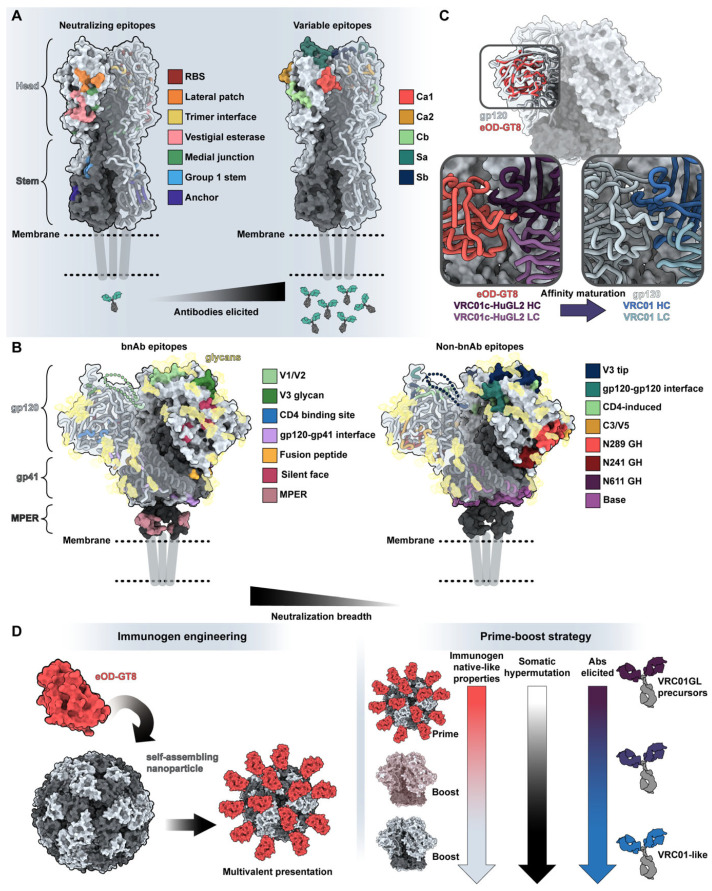
Structure guides vaccine design. (**A**) Neutralizing and variable epitopes of influenza’s H1 hemagglutinin protein (PDB: 7T3D) [91]. Neutralizing epitopes on the left are colored based on a distance of <5 Å between residues of known antibodies and H1 HA (RBS with CH65, PDB: 5UGY [92]; lateral patch with 2B05, PDB: 7T3D [91]; trimer interface with FluA-20, PDB: 6OC3 [93]; medial junction PDB: 8TP9 stem with CR6261, PDB: 3GBN [94]; anchor with 222-1C06, PDB: 7T3D [91]; vestigial esterase domain with H5-specific antibody H5M9, PDB: 4MHH [95]). Immunodominant head epitopes associated with high variability and narrow neutralization breadth are shown on the right [96]. (**B**) Epitopes associated with broadly neutralizing versus limited neutralization breadth on HIV-1’s Env glycoprotein in the prefusion (surface representation of BG505-SOSIP, PDB: 6VFL) [97] and CD4-bound (ribbon representation of B41 SOSIP.664 bound to soluble CD4, PDB: 5VN3) [98] states. Broadly neutralizing epitopes on the left are colored based on a distance of <5 Å between residues of known antibodies and Env (V1/V2 with PG9, PDB: 7T77 [88]; V3 with 10-1074, PDB: 7UCG [99]; CD4 binding site with 3BNC117, PDB: 4JPV [100]; gp120-gp41 interface with 8ANC195, PDB: 5CJX [101]; fusion peptide with ACS202, PDB: 6NC2 [102]; silent face with SF12, PDB: 6OKP [103]; and MPER with 10E8, PDB: 4G6F [104], and 6VPX [105]). Epitopes that generate antibodies without neutralizing breadth are depicted on the right. For simplicity, glycans are depicted as transparent gold surfaces and are not highlighted based on engagement by antibodies. (**C**) Comparison of germline-targeting immunogen eOD-GT8 (PDB: 5IES) [106] with BG505-SOSIP (PDB: 6VFL) [97]. Insets indicate the differences in antigen engagement by VRC01-class precursor antibody VRC01-cHuGL2 (PDB: 5IES; left) [106] and bnAb VRC01 in complex with gp120 (PDB: 3NGB; right) [107]. (**D**) General overview of germline-targeting immunization from immunogen design (left) to overall prime-boost strategies (right). Notably, germline-targeting immunogen eOD-GT8 was designed to bind with high affinity and breadth to germline-reverted antibodies and displayed on a multivalent nanoparticle, the 60-mer lumazine synthase (PDB: 1NQU) [108], to increase immune trafficking and engagement of rare B cells [109].

Understanding the structure of antibody-antigen interfaces, molecular dynamic constraints, and consequences of antibody maturation are crucial for the development of next-generation vaccines. The established method of vaccine development, which involves introducing native antigens to the immune system through inactivated or weakened pathogens, proves inefficient for developing protective immunity against challenging pathogens like influenza viruses and the human immunodeficiency virus (HIV). Due to genomic heterogeneity, high evolution and mutation rates, and competing immune responses of varying quality (Figure 4A,B), wild-type immunogens from these pathogens are insufficient at eliciting broadly protective antibody responses. 

In a process termed “rational vaccine design” or “structural vaccinology”, high-resolution structural information about antibody–antigen interactions is leveraged to design immune-focusing vaccines [76,77,78]. Pathogens that escape immune protection utilize variable epitopes on their surface proteins to distract antibody responses away from conserved and neutralizing sites. By leveraging crucial interactions of broadly neutralizing or protective antibodies as well as identifying interactions that allow pathogens to overcome these responses, tailored immunogens can train the immune system to home in on the most vulnerable sites of the pathogen. 

For example, the hemagglutinin (HA) surface protein of influenza viruses has high sequence variation in the head region but considerable conservation in the stem region [79,80]. The head is significantly more immunogenic, drawing away antibody responses from the conserved stem (Figure 4A). To elicit protective antibody responses, a vaccine will need to redirect immune responses to the stem region. One successful strategy involves focusing antibody responses to the stem region through chimeric HA vaccination in human subjects: by successive immunization of HAs with the same stem region but exotic head regions, broadly-reactive stem antibody responses were recalled and matured [81,82]. Another effective approach used nanoparticles displaying just the stem region to induce broadly reactive stem antibodies [83,84,85,86]. 

Sometimes, additional specificity to particular epitopes and even targeting of germline genes are needed to elicit protective antibody responses. Due to high mutation rates and immunodominance in the Envelope glycoprotein (Env) of HIV (Figure 4B), only highly specific lineages of antibodies will provide protection. Iterative vaccinology and structural comparison of antibodies to Env (Figure 4C) led to the design of epitope-specific immunogens that guide specific precursors of protective antibodies, a method known as “germline targeting” [87,88,89]. Immunization strategies often include priming with germline-targeting immunogens, followed by boosts by immunogens with increasing structural/sequence similarity to native Env epitopes. This strategy intends to increase the somatic hypermutation found in the B-cell repertoire and the neutralizing behavior and breadth of elicited antibodies with each successive boost.

Indeed, in a landmark clinical trial, an immunogen (eOD-GT8, Figure 4D) displaying a single epitope on a scaffold with a masked antigenic surface resulted in the engagement of broadly neutralizing antibody (bnAb) precursor B cells [90]. Through sequential boosting, the desired antibody lineage increased in somatic hypermutation and affinity.

Obtaining high-resolution structures of broadly protective antibodies in complex with antigens is crucial for advancing the development of next-generation vaccines. Research into the interplay between antibody structure, function, dynamics, and immunogenetics will not only inform iterative vaccine design but also be leveraged to engineer novel antibody therapeutics.

## 6. State-of-the-Art Experimental Structure Determination

Structural characterization of protective antibodies bound to their cognate antigen is one of the major research goals of rational vaccine design and vaccinology efforts [110,111]. High-resolution structures are key to understanding the molecular basis of antibody-antigen recognition and thereby complementing other biochemical and biophysical characterizations. These empirical structures are essential in providing unparalleled insights into the mechanisms underlying neutralization and the interactions that contribute to its breadth and potency [112]. The three major structural techniques—NMR spectroscopy, X-ray crystallography, and cryo-electron microscopy (cryo-EM)—remain the most common and widely used methods for epitope mapping at an atomic level. Of these three techniques, X-ray crystallography is the most well established, providing structural details at atomic resolution to elucidate antibody recognition [113]. Up to now, the majority of structures available in the Protein Data Bank (PDB) [114] are crystal structures, emphasizing the tremendous contributions of X-ray crystallography to the field [115]. Crystallography has enabled invaluable structural insights to assess antibody–antigen recognition, characterize antibody affinity maturation, and laid the foundation for antibody structure prediction [116]. A pre-requisite for high-resolution crystal structures are high-quality crystals that diffract, which often requires significant amounts of time and resources. Moreover, structures obtained in the crystalline state may not accurately capture the dynamic behavior of a complex due to crystal packing constraints. 

NMR spectroscopy has been extensively used for the biochemical characterization of protein–protein interfaces [117], including antibody–antigen complexes [118]. In a classic NMR epitope mapping experiment, the antigen is isotopically labeled. Upon mixing the antigen with the antibody of interest, NMR signals will shift from residues in contact with the paratope relative to the signals of the free antigen, thus providing information on the epitope [119]. Key advantages of NMR are its ability to provide dynamic information on antibody-antigen binding and that the samples are studied in solution at near physiological conditions. Unlike X-ray crystallography, NMR spectroscopy can provide an ensemble representation of the protein or complex of interest, which takes into account its inherent flexibility and dynamic behavior in solution [120]. By analyzing the chemical shifts and nuclear Overhauser effects (NOEs) of a protein or complex in solution, NMR data can reveal information about its secondary and tertiary structure as well as its conformational ensemble. This information is critical for understanding the function and stability of proteins and can inform protein engineering efforts. Still, NMR has relatively low throughput for two central reasons: (1) samples require isotopic enrichment, and (2) users need extensive and time-consuming training for successful data collection and processing. Furthermore, NMR spectroscopy is limited to studying only small-sized complexes due to molecular tumbling constraints [121]. This size constraint often necessitates the use of specialized NMR techniques, such as TROSY, to enable the study of larger molecular complexes.

Cryo-EM is fast emerging as a relatively high-throughput structural technique that can study proteins in physiologically relevant solutions to and investigate proteins and complexes between ~100 and 1000 kDa. Moreover, cryo-EM has fewer sample requirements for successful structure determination than X-ray or NMR [122]. Largely owing to the “resolution revolution” and further advancements in data collection technologies—which brought the cryo-EM field from “blobology” to high resolution—have facilitated the relatively routine acquisition of 3–4 Å resolution structures, and in many cases even higher [123,124]. 

Further, the dynamic behavior, on a global or domain-level scale, of antibody complexes can be readily and often routinely assessed using cryo-EM. Moreover, implementing a data processing technique called focused classification can provide high-resolution details of epitope-paratope interfaces. In addition to determining high-resolution structures, the single-particle nature of EM allows the processing of heterogeneous samples, which has been leveraged to perform negative stain electron microscopy polyclonal epitope mapping (nsEMPEM). This method uses antigens as bait in Fab-digested or full IgG serum or plasma samples to purify polyclonal antigen-antibody complexes from vaccinated or infected animals or human participants. After imaging and processing, specific epitopes targeted by a polyclonal antibody response can be determined [125], thereby enabling a relatively complete mapping of the immunogenic landscape of an antigen as well as allowing the temporal tracking of an individual’s immune response by sampling over time. These data can be expanded using cryo-EMPEM to obtain high-resolution data on polyclonal antibody responses. When combined with B-cell repertoire data, cryo-EMPEM can potentially identify and discover novel antibodies by predicting antibody sequences from cryoEM density maps [126]. CryoEMPEM does require intensive sample preparation as well as extensive computational resources for data processing.

## 7. Antibody Engineering—Design of Special Formats/Interface Characterization

### 7.1. The Role and Organization of Constant Domains

In the mid-twentieth century, it was common belief that the variable and constant domains of antibodies act independently [127]. This was supported by genetic studies, which demonstrated that an immunoglobulin is encoded by different gene segments: V(D)J genes carry variable region information while the C segment encodes the constant domain [128]. However, this hypothesis was soon challenged, and later studies proved that the constant domains can affect the affinity and specificity of an antibody towards its antigen [129,130]. For instance, according to the model proposed by Huber et al., the antigen binding causes a stiffening of the antibody molecule, with consequent folding of the hinge peptide, thus facilitating the C_H_1-C_H_2 interaction [131]. Further, the constant regions determine the Ig isotype, which plays a central role in defining the binding affinity to the antigen due to conformational changes of the C_H_1 domains [127,132,133]. Therefore, Ig-like constant domains are of interest since they bind to effector cells, which activate the immune response, and they contribute to binding specificity [20,127,134,135].

As the name “constant domains” already suggests, these domains are evolutionarily more conserved compared to the variable domains. In addition to disulfide-bridged cysteines, residues forming a hydrophobic core within the two sheets are highly conserved, as they contribute to the stabilization of the fold [136,137]. Despite the Ig-like constant domains sharing a similar fold and structure, they differ in their interface properties and dimer formation mechanisms. In fact, the C_H_3-C_H_3 interface is dominated by electrostatic interactions, whereas hydrophobic contacts are mainly responsible for stabilizing the C_H_1-C_L_ interface [72,138]. Both of these interfaces show surprisingly fast interdomain movements, though the C_H_1-C_L_ dimer exhibits higher variability in the interface angle compared to the C_H_3-C_H_3 dimer [13,72]. Only the C_H_2-C_H_2 domains do not directly interact with each other but are instead spatially separated by oligosaccharides. The glycans anchored on the Asn297 residue located on both C_H_2 domains are fundamental to holding the two domains apart and maintaining the characteristic horseshoe shape of the Fc fragment, which is the favorite conformation for interacting with the Fc receptor (Figure 1A) [139,140]. 

### 7.2. Engineering Techniques Led to New Antibody Formats 

Nowadays, monoclonal antibodies (mAbs) are clinically significant molecules, and the hybridoma technology is well established for their industrial production [141,142,143]. Currently, ~175 antibody therapeutics are under regulatory review or on the market, and 13 have been approved in either the USA or Europe in 2022 alone, including four bispecific antibodies [1]. The early financial and clinical success of antibodies increased the curiosity of various pharmaceutical companies and research groups. Endeavours to advance the characterization of these biomolecules and engineer innovative and better-performing biotherapeutics followed soon. Antibody engineering can meet diverse needs, including, avoiding immunogenic responses and rejection in vivo, reducing the molecular weight to facilitate the antibody’s penetration in the cell, improving antibody developability, addressing novel therapeutic needs, promoting large-scale production, and decreasing costs. Here, we will provide a general overview of the most common engineering techniques and describe how new antibody formats accommodate pharmaceutical demands.

### 7.3. Humanization

Several engineering studies attempted to address the strong immunogenic reaction and the undesired anti-drug antibody (ADA) response caused by repeated administration of mAbs [144]. The origin of the ADA response is not fully understood, but experts hypothesize it may be caused by the murine nature of the mAbs [145]. Therefore, several studies aimed to develop humanized antibodies and avoid the human anti-mouse antibody response (HAMA) [146,147]. To achieve this goal, transgenic mice featuring a human immunoglobulin gene repertoire are used [148,149,150]. Generally, humanization describes the engineering effort of making a non-human antibody more human-like, i.e., by reducing as much of the murine content as possible while sustaining affinity and specificity [151,152]. There are several strategies to achieve antibody humanization. A well-established technique is the production of chimeric antibodies, where the non-human constant domains are exchanged with human constant domains and the non-human and antigen-specific variable domains are unaltered [153]. Another common method to obtain humanized Fabs is CDR grafting, which consists of grafting non-human CDR loops onto a human Fab framework [154,155,156,157,158]. In order to avoid decreased affinity, some non-human residues in the framework region are retained; these are the Vernier-zone residues, which can affect the CDR conformation and the binding affinity [7,159]. 

### 7.4. Affinity Maturation

Affinity maturation describes a crucial and complex process in the adaptive immune system where antibodies with higher affinity are produced in response to an antigen [160]. Thereby, B-cells adapt their receptors towards the epitope through multiple rounds of somatic hypermutation events followed by selection occasions in germinal centers. This process can increase the affinity of a naïve B-cell receptor (BCR) towards a target antigen up to a thousand-fold. Engineering strategies aim to mimic the affinity maturation process to modulate antibody affinity and specificity [160,161]. Interestingly, residues that are mutated during this process are not solely located in the CDR loops but can also be part of the framework region [162], showcasing the high complexity of predicting relevant amino acids that determine/govern affinity. Various studies have succeeded in improving antibody affinity by using combinatorial libraries [163] or computational design [164,165,166,167,168]. Additionally, high-resolution structural characterization enables the elucidation of the structural consequences of affinity maturation and the identification of key residues contributing to this increase in affinity and specificity [169,170]. Based on these empirical structures, long-timescale MD simulations have been used to provide a mechanistic understanding of the structural and dynamic changes in different stages of affinity maturation. It has been demonstrated that affinity maturation does not only result in a rigidification of the antigen-binding site but also restricts the flexibility of the elbow and interface angles in the Fab [13,169]. The observed rigidification corresponds to a reduction of energy minima, shifting the probability towards the binding competent conformation and favoring the lock-and-key binding mechanism [171,172]. Conversely, germline antibodies follow the conformational selection paradigm, as they adopt multiple weakly populated conformational states that can recognize different antigens [173,174,175]. Upon binding to a specific antigen, the respective state probabilities subsequently shift towards the binding competent state. 

### 7.5. Bispecific Antibodies

**Figure 5:**
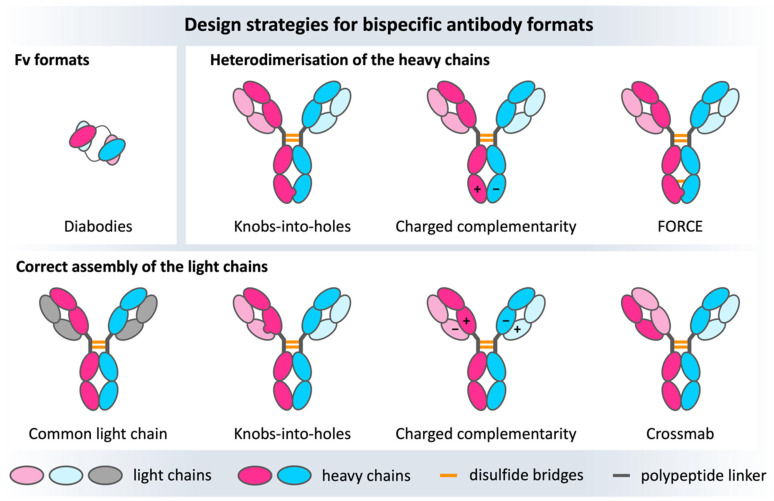
Overview of some of the numerous bispecific design strategies for bispecific antibodies. The heavy chains are colored bright pink/blue, the light chains are depicted in light blue/pink and gray. The upper row shows Fv formats focusing on engineering and optimizing the V_H_-V_L_ interface on the left, as well as heavy chain heterodimerization approaches (CH_3_-CH_3_ complementarity), whereas the lower row represents the light chain heterodimerization strategies concerning mainly the C_H_1-C_L_ interface.

Bispecific antibodies are the products of tremendous advances in antibody engineering, resulting in a molecule that can simultaneously recognize two different epitopes and/or bring two targets in close proximity. The most prominent bispecific antibody designs are full-size and IgG-like, in which the C_H_3-C_H_3 domains are engineered to favor heavy chain heterodimerization over homodimerization (Figure 5). The idea behind this antibody format is that the resulting IgG-like molecule is formed by two different heavy chains and two different light chains and consequently offers distinct antigen binding sites. The main challenge in engineering and producing bispecific IgG-like antibodies is the high number of pairing possibilities resulting from two different heavy and light chains. The potential combinations can lead to several unwanted side products [176,177,178]. Nowadays, there are several design strategies that modify domain interfaces by introducing point mutations to ensure the desired heterodimeric interface is formed (Figure 5). The first engineering steps focus on optimizing the association of the heterodimeric heavy chains, which can be achieved by establishing charge interactions or increasing steric complementarity. Charge complementarity can be realized by inserting opposite charged residues in the C_H_3-C_H_3 interface [179,180,181], while steric complementarity has been obtained with the knobs-into-holes (KiH) technology. In the KiH approach, a bulky tryptophan residue is introduced on one chain to fit into a designed hydrophobic pocket on the other chain, resulting from three mutations [182,183]. Starting from the KiH method, the expression yields of the heterodimers improved thanks to the format chain exchange (FORCE) technology, in which the final product is further stabilized by a disulfide bridge [184]. Apart from optimizing the heavy chain heterodimerization, the correct light chain pairing must be ensured to obtain two functional and distinct antigen binding sites. The most obvious solution is using a common light chain; however, this strategy restricts the accessible combinations/paratopes [185,186,187]. Alternatively, the C_H_1-C_L_ interface can be engineered following the same strategies used for the C_H_3-C_H_3 interface, resulting in the correct heavy-light chain pairing [188]. With the more recent Crossmab technology developed by Roche, correct association of the light chain with the respective counterpart can be enabled by exchanging heavy and light chain domains within one Fab, thereby retaining antigen-binding affinity while preventing light chain mispairings [189].

These tremendous antibody engineering successes are reflected in the numerous bispecific antibodies that are already approved on the market or are currently in late-stage development [1].

### 7.6. Small Size Antibody Formats

Natural IgGs have a weight of ~150 kDa, and their large size often represents a challenge for their production and administration. Therefore, smaller molecules that retain the binding affinity and specificity of the original antibody but have more favorable expression and pharmacokinetic properties have been pursued. Well-established examples of these small antibody formats are Fabs or single-chain variable fragments (scFv), the latter of which consists of a variable fragment of a heavy chain and one of a light chain, joined by a flexible peptide linker. The length of the linker (~15 residues) is fundamental to obtaining the correct folding, and its amino acid composition can be engineered to increase the affinity and specificity of the scFv [190,191]. Another antibody format is the “diabody”. Diabodies are bivalent antibodies constructed by connecting two Fv domains via a short peptide linker consisting of three to twelve residues. This linker is too short to allow mispairings of the two Fvs, and, therefore, the domains form two binding sites [192]. The two sites of a diabody can be either identical, resulting in a monospecific biologic, or distinct, resulting in bispecificity [193,194]. 

## 8. Special Formats from Natural Sources

**Figure 6:**
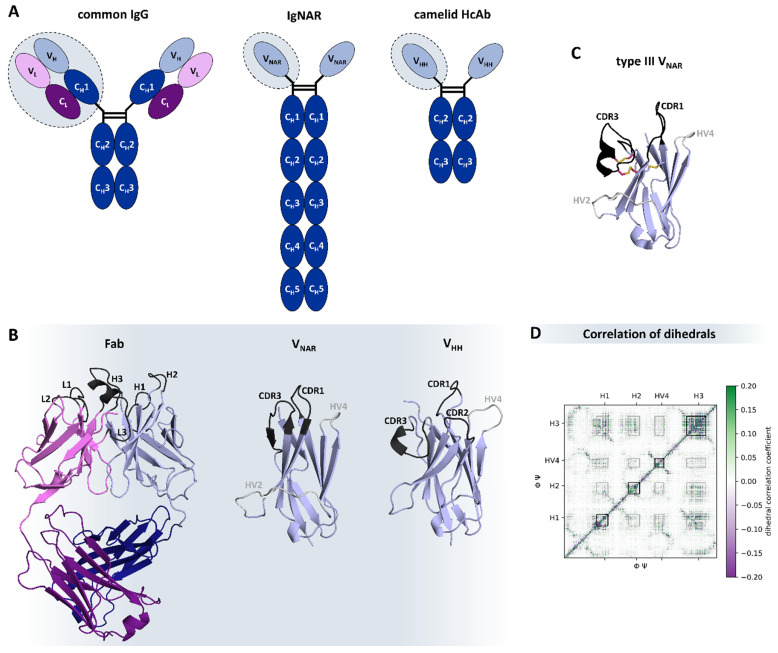
Natural occurring heavy-chain only antibodies in comparison to common IgG isotypes: (**A**) Differences in antibodies in humans, sharks, and camelids as shown by their schematic structures. The heavy chains are colored blue, while the light chains are purple. The variable domains are shown in lighter shades. (**B**) Structural differences of the antigen binding relevant sections: Fab (PDB: 6MAM) [232], VNAR (type 4, PDB: 4HGK) [213], and VHH (PDB: 2P49) [233], respectively. The CDR loops are colored black, and the hypervariable regions are gray. In (**C**), the disulfide bridges of a type III VNAR (PDB: 1SQ2) [196] are highlighted. Depending on the number of additional disulfide bridges and distinct conserved residues, shark VNARs can be grouped into four different types [196,199,213]. The pink-colored residues point out the additional bonds in this exemplary system. (**D**) Exemplary correlation of the backbone torsions of a nanobody simulation. Green depicts a positive correlation coefficient and purple a negative one. Besides the diagonal, which represents the correlation of adjacent residues, a clear influence of the different binding relevant regions (CDR-loops: H1, H2, and H3, as well as HV4) is highlighted, describing the correlated movements involved in shaping the paratope.

In contrast to the conventional IgG isotype, there are different types of naturally occurring antibody formats found in species like cartilaginous fish, e.g., sharks, and Camelidae, e.g., camelids. These special formats, also known as heavy-chain only antibodies (HcAb), are deprived of the light chains and therefore characterized by the assembly of only two heavy chains [195,196]. While the Immunoglobulin New Antigen Receptor (IgNAR) found in cartilaginous fish is comprised of one variable (VNAR) and five constant domains, the Camelidae variant has one variable (V_H_H, or nanobody) and two constant domains (Figure 6A) [197,198]. There are further structural differences, especially in the variable domains, which represent the functional and structural analogues of the Fab in conventional antibodies and are critical for antigen-binding (Figure 6B). First, the VNAR and the VHH are much smaller than a whole Fab (consisting of 4 domains) and, hence, are able to wend into tissue in a fast and specific way [199,200]. In this context, it has been shown that VNARs can serve as shuttles for larger proteins to cross the blood-brain barrier [201,202]. Both single-domain antibodies have a characteristically prolonged CDR3 loop that has higher variability in sequence and structure than the CDR-H3 in conventional IgGs [203,204]. The consequently convex-shaped paratope is able to bind into cavities and cryptic epitopes [205,206]. However, the high conformational flexibility of HcAbs can result in a high entropic cost upon antigen binding, which led to the evolution of additional cysteine bonds to rigidify and stabilize the binding site (Figure 6C) [207]. Nevertheless, it is still unclear what precise role rigidity plays in the antigen binding process [208]. The overall high stability of HcAbs arises from the challenging conditions they evolved in, e.g., the high urea concentration in the blood of sharks, which has a denaturizing effect [197,209]. Furthermore, the increased number of solvent-exposed hydrophilic residues in the HcAbs contributes to their good solubility in aqueous solutions and is required to prevent the formation of a heavy-light chain interface, which is mainly mediated by a hydrophobic core [72,210]. For VNARs, this characteristic property cannot be assigned to specific residues, while for nanobodies, this is mainly dependent on four residues in framework 2 (F42, E49, R50, and G52 in IMGT numbering) [198]. Furthermore, VNARs are missing the CDR2 loop, which is replaced by a hypervariable region with hydrophilic character that forms a belt-like conformation, from which side chains point into the alleged V_H_-V_L_ interface [211]. The hypervariable region 4-loop (HV4-loop) is of special importance for all HcAbs since it influences antigen-binding directly and indirectly by stabilizing distinct paratope conformations [170], as it was previously demonstrated for T-cell receptors [212]. In addition, all loops collude with each other and support the hypothesis of a complementary evolution (Figure 6D) [200].

The greatest difference between nanobodies and VNARs lies in their sequences. VNARs evolved around 500 million years ago and therefore show high sequence dissimilarities to vertebrates yet astonishingly high structural resemblance to κ-light chain variable domains [197,213,214,215]. VHHs, on the other hand, are more related to other vertebrate heavy chain variable domains since they evolved only 40 million years ago [216,217]. Although they share a low sequence similarity, the structural likeliness of the HcAbs to other heavy chain variable domains is remarkable, which highlights their coherent evolution [218,219]. This insight facilitates the engineering efforts of nanobodies and reduces the risk of immunogenicity in comparison to VNARs [220,221]. In fact, various studies have focused on designing and optimizing these single-domain antibodies to exploit their advantageous features for future biotherapeutics.

In addition to their unique sequence and structural features, understanding the consequences of affinity maturation and point mutations on their binding properties, i.e., affinity and specificity, is of utmost importance to enable modulation and fine-tuning of their function [222,223]. Indeed, structural and dynamic characterizations of nanobodies have, on the one hand, revealed residues that play a crucial role in antigen recognition and, on the other, identified that an increase in affinity and specificity can be accompanied by a rigidification of the antigen binding site [170]. Apart from the investigation of the antigen binding properties, there are ongoing studies about the humanization strategies of HcAbs, which are complicated for shark VNARs due to their low sequence similarity [213,221,224]. Nevertheless, there are both nanobodiesy and VNAR drug candidates in clinical trials [225,226,227]. Considering the beneficial features and improvements in designing and engineering HcAbs, predicting their structure, in particular the conformations of CDR loops, still remains challenging. The extended CDR3 loop conformation is especially difficult to predict accurately due to the high diversity in length, sequence, and structure. Additionally, the resulting high conformational variability suggests that one single static structure might not be sufficient to capture the high conformational diversity of this loop [228,229]. However, in addition to a structural and dynamic characterization of these novel antibody formats, various tools are emerging to address these challenges based on deep-learning models [44,230,231]. 

## 9. Conclusions

The numerous high-quality antibody structures available in the PDB (originating from X-ray crystallography, cryo-EM, and NMR spectroscopy) provide an atomic view of the affinity maturation process, influence engineering strategies, and advance the understanding of antigen binding mechanisms. In addition, these structures, in combination with neural networks, substantially improve state-of-the-art antibody structure prediction tools. These models can, however, still suffer from structural and physical inaccuracies and should be carefully reviewed. The CDR loops especially remain challenging to accurately predict due to their high conformational diversity. Thus, capturing the high variability of the CDR loops is another critical aspect for informing antibody engineering efforts and improving structure prediction algorithms. In particular, processes such as aggregation and stability are strongly governed by dynamics, highlighting the importance of considering antibodies as conformational ensembles in solution to guide rational antibody design. The tremendous advances in antibody engineering, focusing on the shape or charge complementarity of the constant domain interfaces, led to various novel formats, even further enhancing the applicability of antibodies as biotherapeutics. Finally, single-domain antibodies, originating from sharks and camelids, represent a highly potent alternative to conventional IgG antibodies, providing numerous applications and design opportunities.

## Data Availability

Not applicable.
